# MicroRNAs as the critical regulators of Doxorubicin resistance in breast tumor cells

**DOI:** 10.1186/s12935-021-01873-4

**Published:** 2021-04-15

**Authors:** Amir Sadra Zangouei, Maliheh Alimardani, Meysam Moghbeli

**Affiliations:** 1 Student Research Committee, Faculty of Medicine, Mashhad University of Medical Sciences, Mashhad, Iran; 2 Department of Medical Genetics and Molecular Medicine, School of Medicine, Mashhad University of Medical Sciences, Mashhad, Iran

**Keywords:** Breast cancer, Chemo-resistance, Doxorubicin, MicroRNA, Chemotherapy

## Abstract

**Background:**

Chemotherapy is one of the most common treatment options for breast cancer (BC) patients. However, about half of the BC patients are chemotherapeutic resistant. Doxorubicin (DOX) is considered as one of the first line drugs in the treatment of BC patients whose function is negatively affected by multi drug resistance. Due to the severe side effects of DOX, it is very important to diagnose the DOX resistant BC patients. Therefore, assessment of molecular mechanisms involved in DOX resistance can improve the clinical outcomes in BC patients by introducing the novel therapeutic and diagnostic molecular markers. MicroRNAs (miRNAs) as members of the non-coding RNAs family have pivotal roles in various cellular processes including cell proliferation and apoptosis. Therefore, aberrant miRNAs functions and expressions can be associated with tumor progression, metastasis, and drug resistance. Moreover, due to miRNAs stability in body fluids, they can be considered as non-invasive diagnostic markers for the DOX response in BC patients.

**Main body:**

In the present review, we have summarized all of the miRNAs that have been reported to be associated with DOX resistance in BC for the first time in the world.

**Conclusions:**

Since, DOX has severe side effects; it is required to distinguish the non DOX-responders from responders to improve the clinical outcomes of BC patients. This review highlights the miRNAs as pivotal regulators of DOX resistance in breast tumor cells. Moreover, the present review paves the way of introducing a non-invasive panel of prediction markers for DOX response among BC patients.

## Background

Breast cancer (BC) is one of the leading causes of cancer related mortalities among females [1]. It is the most frequently occurring female malignancy which is responsible for nearly 31% of all cancers diagnosed in women. An estimated 1,200,000 newly diagnosed BC patients and 465,000 mortality are reported annually in the world [2]. BC can be classified to the various distinct histological types such as lobular, ductal, papillary, and tubular carcinomas [3]. It can be also classified according to the immuno-pathological features such as progesterone receptor (PR), HER2, and estrogen receptor (ER) expressions [4]. Triple-negative breast cancer (TNBC) accounts for almost 15–20% of all BC cases which is referred to any breast tumor lacking the expressions of ER, PR, and HER2 [5, 6]. Chemotherapy is a routine treatment option for BC, while almost half of the initially responsive tumors develop resistance to various chemotherapeutic regimens [7].

Adriamycin (ADR) or Doxorubicin (DOX) is regarded as the most effective chemotherapeutic medication used for BC treatment; however, DOX effectiveness is negatively affected by multidrug resistance in BC cells during chemotherapy [8]. About 30–50% of metastatic BC patients are responsive to the DOX treatment [9]. DOX as a topoisomerase II inhibitor suppresses tumor growth through DNA replication interfering [10]. Multidrug resistance (MDR) affects the efficacy of cancer therapy and is responsible for treatment failure, tumor progression, and recurrence in a large number of BC patients. Deregulation of drug efflux transporters such as ABCB1 and multiple resistance protein-1 (MRP1) are important factors associated with MDR. Abnormal increased DNA repair processes, drug detoxification, and aberrant expression of oncogenes and tumor suppressors, are also other driving forces behind the MDR development [11, 12]. Mechanisms of DOX resistance can be classified into: (1) up regulation of drug-resistant proteins and membrane multidrug pumps in cancer cells, and (2) disruption in cellular signaling pathways which leads to the suppression of the apoptosis induced by DOX.

MicroRNAs (miRNAs) are small non-coding RNAs with 9–22 nucleotides length serving as post-transcriptional regulators of gene expression via binding to the 3′-untranslated region (UTR) of their target mRNAs that results in mRNA degradation or translational suppression [13]. Dysregulation of various miRNAs have been reported to be associated with the tumor progression and drug resistance [14–16]. Inhibition of miRNA activity by competitive inhibitors including miRNA sponge or target mimic has been used to study their functions. Sponge miRNAs can bind with a non-coding transcript or 3′ UTR of target gene which are expressed by U6 or CMV promoters. Lentiviral and retroviral vectors with sponge RNAs have continuous miRNA suppression in either dividing or non-dividing cells [17]. There is not any efficient method to distinguish the non-responders from those who will respond to chemotherapy. Therefore, a reliable approach for classifying patients in order to prevent unwanted side effects of chemotherapy and optimize the treatment outcome is imperative. Regarding severe DOX side effects, it is required to clarify the molecular mechanisms involved in DOX resistance to provide novel efficient therapeutic modalities to improve the clinical outcomes of BC patients. Since, microRNAs are non-invasive and more stable factors compared with mRNAs, they can be introduced as efficient and reliable markers of DOX response in BC patients.

In the present review, we have summarized all of the miRNAs that have been reported to be associated with DOX resistance in BC for the first time in the world (Table 1). We categorized the reported miRNAs based on their targets to clarify the molecular mechanisms of miRNAs mediated DOX resistance in breast tumor cells.

**Table 1:** All of the miRNAs associated with Doxorubicin resistance in BC

Study	Year	Gene	Country	Target	samples	Results
Developmental factors and signaling pathways						
Wu [24]	2019	miR-140-5p	China	WNT1	MCF-7, MDA-MB-231 cell lines	Increased Dox sensitivity
Cheng [31]	2019	miR-137	China	FSTL1	87 patients HCC38, MDA-MB-231, and MDA-MB-468 cell lines	Increased Dox sensitivity
Xiong [36]	2018	miR-613	China	DAAM1	123 patients MDA-MB-231, MCF-7, HEK-293 T, and SUM1315 cell lines	Increased Dox sensitivity
Li [42]	2012	miR-34a	China	NOTCH1	38 patients MCF-7 cell line	Increased Dox sensitivity
Hu [47]	2016	miR-760	China	NANOG and SNAIL	MCF-7, MDA-MB-231 cell lines	Increased Dox sensitivity
Kim [55]	2016	miR-34a	Korea	PRKD1	MCF-7, MDA-MB-231 cell lines	Increased Dox sensitivity
PI3K/AKT and MAPK signaling pathways						
Shen [57]	2016	miR-29a	China	PTEN	MCF-7 cell line	Increased Dox resistance
Hu [58]	2016	miR-205	China	VEGFA and FGF2	30 patients MCF-7 cell line	Increased Dox sensitivity
Liu [59]	2019	miR-202-5p	China	PI3K and AKT	62 patients MCF-10A and MCF-7 cell lines	Increased Dox resistance
Kopp [70]	2012	miR-200c	Germany	TRKB and BMI1	MDA-MB-436 and BT474 cell lines	Increased Dox sensitivity
Xie [73]	2018	miR-132 and miR-212	China	PTEN	53 patients MCF-7 cell line	Increased Dox resistance
Shen [74]	2017	miR-222	China	PTEN	MCF-7 cell line	Increased Dox resistance
Wang [75]	2011	miR-21	China	PTEN	MCF-7 cell line	Increased Dox resistance
Chu [76]	2017	miR-93	China	PTEN	16 patients MCF-7 cell line	Increased Dox resistance
Chen [77]	2013	miR-200c	China	ZEB1	MCF-7 cell line	Increased Dox sensitivity
Fang [84]	2014	miR-30c	China	YWHAZ	MCF-7, MDA-MB-231 cell lines	Increased Dox sensitivity
Du [86]	2019	miR-137	China	DUSP4	MCF-7, HCC1937, and MDA-MB-468 cell lines	Increased Dox sensitivity
Mi [88]	2018	miR-381	China	FYN	MCF-7, MDA-MB-231 cell lines	Increased Dox sensitivity
Zhao [92]	2016	miR-302	China	MEKK1	MCF-7 cell line	Increased Dox sensitivity
Apoptosis, cell cycle, and DNA repair						
Zheng [96]	2016	miR-181b	China	BIM	30 patients MCF-10A, T-47D, MCF-7, MDA-MB-231, and MDA-MB-435 cell lines	Increased Dox resistance
Dai [97]	2019	miR-222	China	BIM	25 patients MCF-7 cell line	Increased Dox sensitivity
Long [101]	2015	miR-193b	China	MCL1	MCF-7 cell line	Increased Dox sensitivity
Hu [103]	2015	miR-218	China	BIRC5	MCF-7 and CAL-51 cell lines	Increased Dox sensitivity
Li [108]	2019	miR-3609	China	PDL1	47 patients HBL-100, MCF-7, MDA-MB-231, and MDA-MB-468 cell lines	Increased Dox sensitivity
Zhang [112]	2019	miR-192-5p	China	PPIA and BCL2	MCF-10A, MCF-7 cell lines	Increased Dox sensitivity
Zhang [114]	2016	miR-214	China	RFWD2	31 patients MCF-7, MDA-MB-231, and MDA-MB-468 cell lines	Increased Dox sensitivity
Tormo [115]	2019	miR-449	Spain	CDK2, E2F1, and E2F3	30 patients MDA-MB-231, MDA-MB-468, and MCF-7 cell lines	Increased Dox sensitivity
Lu [121]	2020	miR-140	China	FEN1	MCF-7 cell line	Increased Dox sensitivity
Lin [124]	2019	miR-30c	China	REV1 and FANCF	MCF-7, ZR-75–1, T-47D, MDA-MB-231, and MCF-10A cell lines	Increased Dox sensitivity
Transporters						
Lu [129]	2015	miR-134	China	ABCC1	40 patients MCF-7 cell line	Increased Dox sensitivity
Chang [131]	2018	miR-199a	China	MRP1	MCF-7	Increased Dox sensitivity
Gao [132]	2016	miR-145	China	MRP1	112 patients MCF-7, MDA-MB-231, MDA-MB-453, MDA-MB-468, and MCF-10A cell lines	Increased Dox sensitivity
Chen [135]	2012	miR-200c	China	MDR1	39 patients MCF-7 and MDA-MB-231 cell lines	Increased Dox sensitivity
Kovalchuk [136]	2008	miR-451	Canada	MDR1	MCF-7 cell line	Increased Dox sensitivity
Hu [137]	2019	miR-124-3p	China	ABCC4	40 patients MCF-7 and MCF-10A cell lines	Increased Dox sensitivity
Yuan [143]	2015	miR-133a	China	UCP2	MCF-7 cell line	Increased Dox sensitivity
TGF-β and JAK/STAT signaling pathways						
Sun [146]	2018	miR-574	China	SMAD4	30 patients MCF-7 cell line	Increased Dox resistance
Jiang [148]	2014	miR-489	China	SMAD3	MCF-7 cell line	Increased Dox sensitivity
Liang [150]	2019	miR-548-p	China	PBLD	MCF-7 and MDA-MB-231 cell lines	Increased Dox resistance
Liu [155]	2019	miR-124	China	STAT3 and HIF1	MCF-7 cell line	Increased Dox sensitivity
Enzymes and structural proteins						
Han [161]	2019	miR-181c	China	OPN	29 patients MCF-7 cell line	Increased Dox sensitivity
Zhang [165]	2019	miR-135b-5p	China	AGR2	28 patients MCF-7 and MDA-MB-231 cell lines	Increased Dox sensitivity
Bolandghamat pour [171]	2019	miR-154	Iran	NAMPT	MCF-7, MCF-10A, and MDA-MB-231 cell lines	Increased Dox sensitivity
Li [175]	2018	miR-770	China	STMN1	MDA-MB-231, MDA-MB-468, and THP-1 cell lines	Increased Dox sensitivity

## Main text

### Developmental factors and signaling pathways

**Fig. 1:**
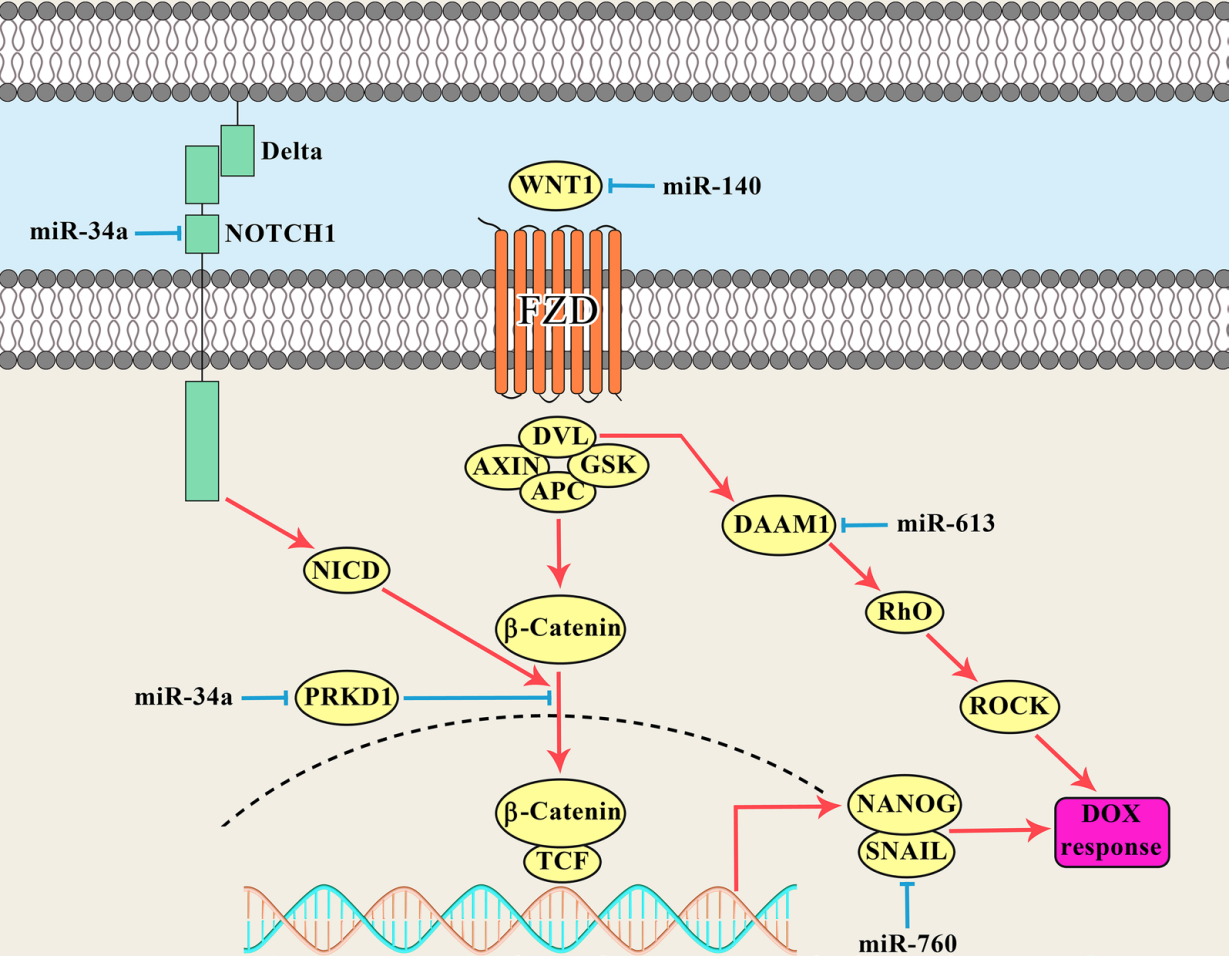
miRNAs are involved in DOX response (resistance or sensitivity) of breast tumor cells via regulation of WNT and NOTCH signaling pathways

Developmental signaling pathways such as WNT and NOTCH have pivotal roles in DOX response of breast tumor cells which can be regulated by miRNAs (Fig. 1). WNT signaling is a developmental pathway triggered by interaction between WNT ligands and Frizzled (FZD) receptors that result in the activation of non-canonical and canonical cascades. WNT family of proteins consists of a variety of cysteine-rich secreted glycoproteins involved in cell proliferation, polarity, apoptosis, DNA repair, embryogenesis, and tumor progression [18–20]. ALDH1 + breast cancer stem cells (BCSCs) are a sub population of tumor cells with a high self-renewal and tumorigenic capacities. MiR-140-5p modulates the BCSCs through inhibiting the self-renewal factors including WNT, SOX2, and SOX9 [21]. OCT4 is also the principal transcription factor for the regulation of pluripotency and self-renewing capabilities in the embryonic stem cells [22]. WNT1 induces tumor cell cycle progression and migration via interaction with specific FZD receptors in the surface of target cells which leads to β-catenin nuclear transportation and activation [23]. It has been reported that miR-140-5p reduced BCSCs proliferation, self-renewal, and sphere-formation via WNT signaling targeting. MiR-140-5p also decreased the levels of OCT4 and ALDH1 expressions and reduced the sphere formation. Moreover, miR-140-5p sensitized BCSCs to DOX mainly through the suppression of WNT1/ABCB1 axis [24].

Integrin β3 belongs to the integral cell-surface receptors that mainly serves as a link between the cytoskeleton and extra cellular matrix (ECM) to regulate cell adhesion, proliferation, migration, angiogenesis, cytoskeletal organization, and tumorigenesis [25–27]. It also enhances the growth factor release, invasion, migration, and epithelial mesenchymal transition (EMT) process in breast tumor cells [28–30]. WNT/β-catenin pathway exerts its effect on intracellular signal transduction via cell surface receptors such as integrin β3. It has been shown that there was FSTL1 up regulation in TNBC samples and cell lines compared with non-TNBC samples and normal mammary epithelial cells, respectively. MiR-137 also inhibited WNT/β-catenin signaling and suppressed stemness and DOX resistance of BC cells through targeting *FSTL1* [31]. Dishevelled-associated activator of morphogenesis 1 (Daam1) is involved in WNT/PCP signaling pathway through interaction with Dishevelled [32, 33]. It is associated with increased cell migration via stimulation of actin reorganization during gastrulation, filopodia formation, and female germ cells meiosis [34, 35]. It has been reported that there was a converse association between the levels of miR-613 expressions and lymph node involvement in BC patients. MiR-613 was involved in regulation of DOX sensitivity via inhibition of Daam1/RhoA pathway [36].

EMT is a biological process allows epithelial cells to lose their polarity and cell–cell adhesion to gain mesenchymal organization. It has pivotal roles in various physiological and pathological processes including embryogenesis, tissue homeostasis, and tumorigenesis [37, 38]. Tumor cells that undergo EMT, acquire stem cell-like properties correlated with malignant behavior and enhanced chemo resistance [39].

Notch signaling is one of the critical developmental pathways involved in cell differentiation, migration, and drug resistance via Notch receptors (Notch1-4) and ligands (DLL and Jagged). This signaling pathway also regulates the EMT especially in cancer stem cells (CSCs) that is a fundamental process in drug resistance and tumor relapse [18, 40, 41]. It has been reported that the miR-34a expression regulated the ADR response in BC cells through *NOTCH1* targeting. There was also significant miR-34a down regulation in MCF-7/ADR cells compared with MCF-7 cells. MiR-34a significantly increased ADR sensitivity. Moreover, ADR responders had higher levels of miR-34a expressions compared with non-responders [42].

Nanog is a developmental transcription factor involved in self-renewal and differentiation of stem cells [43, 44]. It is also a critical factor for the regulation of EMT process and chemo resistance during tumor progression [45, 46]. It has been reported that there was significant miR-760 down regulation in MCF-7/DOX and DOX resistant BC tissues in comparison with MCF-7 cells and chemo sensitive tissues. MiR-760 increased DOX sensitivity in BC cells through NANOG inhibition and also reversed EMT by SNAIL down regulation and E-cadherin up regulation in MCF-7/DOX cells [47]. CSCs are a small subset of tumor cells with self-renewal, recurrence, and chemo resistance capabilities [48]. Various miRNA are implicated in the formation of BCSCs and self-renewal maintenance [49]. MiR-34c was shown to inhibit the EMT process and decrease the self-renewal capabilities of BCSCs [50]. Serine/threonine-protein kinase D1 (PRKD1) is a downstream effector of diacylglycerol and protein kinase C that mediates the function of growth factors, hormones, and neurotransmitters [51]. It is also involved in activation of NF-kB signaling, DNA synthesis, and cell cycle progression [52–54]. PRKD1 enhanced the self-renewal ability of BCSCs via the GSK3/β-catenin signaling pathway. MiR-34a targeted the *PRKD1* and reduced breast cancer stemness through the GSK3/β-catenin signaling axis [55].

### PI3K/AKT and MAPK signaling pathways

**Fig. 2:**
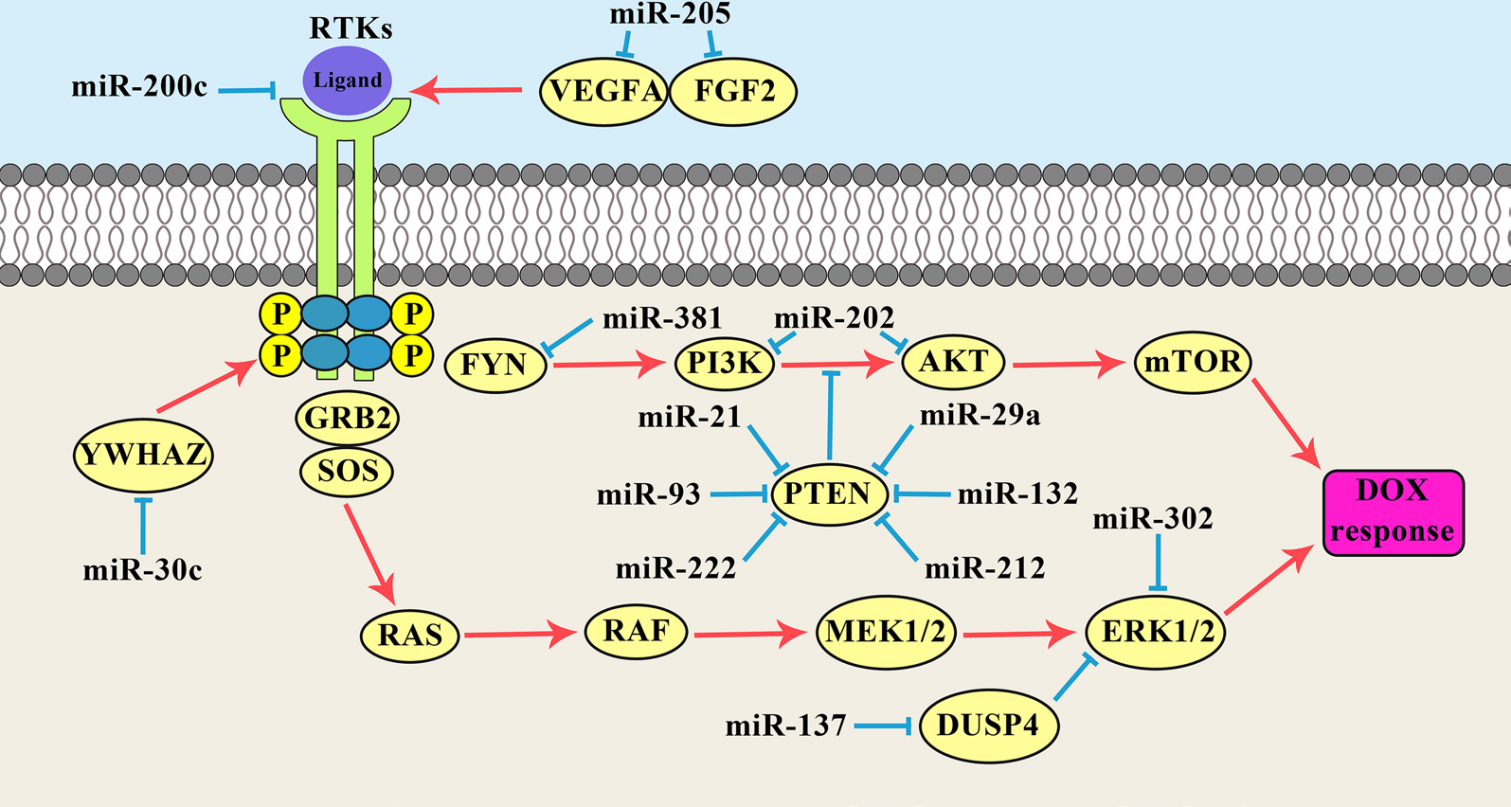
miRNAs have important roles in DOX response (resistance or sensitivity) of breast tumor cells via regulation of PI3K/AKT and MAPK signaling pathways

MiRNAs are involved in regulation of DOX response in BC through PI3K/AKT and MAPK signaling pathways (Fig. 2). The PI3K/AKT pathway has critical roles in regulation of cell proliferation and tumor progression. PI3K activates AKT that regulates various effectors such as CREB, p27, FOXO, and mTOR. Tyrosine kinase receptors and phosphatase and tensin homolog (PTEN) are known as the positive and negative regulators of the PI3K/AKT pathway, respectively. Glycogen Synthase Kinase 3β (GSK-3β) is a serine/threonine kinase involved in the PI3K/AKT signaling pathway [56]. It has been reported that miR-29a up regulation was associated with the p-AKT and p-GSK3β over expressions which promoted the DOX-resistance in breast tumor cells [57]. MiR-205 up regulation was significantly associated with sensitivity to TAC (docetaxol, doxorubicin plus cyclophosphamide). There were miR-205 down regulations in drug-resistant BC cell lines, however, ectopic expression of miR-205 resulted in DOX restoration and taxol sensitivity via inducing apoptosis in both of the aforementioned drug-resistant BC cells. Moreover, miR-205 suppressed the PI3K/AKT signaling by VEGFA and FGF2 down regulations which resulted in enhanced tumor cell apoptosis upon chemotherapy [58]. Another study showed that there were significant miR-202-5p up regulations in DOX resistant BC specimens and cell lines. MiR-2020-5p enhanced breast tumor cell proliferation and DOX-resistance through the PTEN/PI3K/AKT signaling pathway [59].

MiR-200c is an important regulator of EMT process through suppressing the E-cadherin transcriptional repressors (ZEB1 and ZEB2) [60–62]. Tropomyosin receptor kinase B (TrkB) is a tyrosine kinase receptor involved in cell differentiation, proliferation, and migration that functions through activation of the PI3K/AKT and MAP kinases [63]. The AKT phosphorylation plays an important role in promoting cell survival via phosphorylating and suppressing pro-apoptotic caspases and Bad [64, 65]. Bmi1 belongs to the polycomb-group protein family involved in self-renewal maintenance and inhibition of senescence [66–68]. It also down regulates the p19Arf that leads to p53 degradation by MDM2 [69]. It has been reported that the miR-200c increased DOX sensitivity via TrkB and Bmi1 inhibitions in breast tumor cells [70].

PTEN is a tumor suppressor that functions as a negative regulator of the AKT pathway, tumor cell migration, and apoptosis [71, 72]. It is a dual-specificity phosphatase that dephosphorylates lipid and protein substrates. It has been reported that there were miR-132 and miR-212 up-regulations in DOX resistant BC tumors and cell lines by PTEN inhibition. The miR-132 and miR-212 up regulations were also associated with NF-κB activation [73]. The FOXO is a family of transcription factors which are the downstream targets of AKT. It has been reported that the miR-222 was correlated with DOX resistance in BC cells through regulation of PTEN/AKT/FOXO1 axis [74]. MiR-21 also regulates the DOX-sensitivity in BC cells through targeting PTEN. There was a significant miR-21 up regulation in MCF-7/DOX cells compared with parental MCF-7 cells. PTEN was significantly suppressed in MCF-7/DOX cells compared to MCF-7 cells. Down regulation of miR-21 promoted the CASP3-mediated apoptosis in MCF-7/DOX cells which may be the possible explanation for increased sensitivity of MCF-7/DOX cells to DOX following transfection of miR-21 inhibitor [75]. It has been shown that there was significant miR-93 up regulation in ductal BC tissues compared with normal margins. MiR-93 markedly increased MCF-7 proliferation and survival after DOX treatment compared with control. Multidrug resistance-related genes (*MDR*, *MRP*, and *BCRP*) were also significantly up regulated in the MCF-7-miR-93 mimic cells. MiR-93 regulated DOX-resistance and EMT in BC cells through targeting PTEN [76]. It has been shown that miR-200c up regulated the E-cadherin through ZEB1 suppression. It also reduced AKT phosphorylation by PTEN up regulation that resulted in increased DOX sensitivity in breast tumor cells [77].

Mitogen-activated protein kinase (MAPK) is a signaling pathway that functions via sequential activation of a MAPK module including MAPKKK, MAPKK, and MAPK. There are various MAPKs such as ERK, JNK, and p38 involving in the cell growth, metabolism, and apoptosis [78]. The p38MAPK signaling has important role in apoptosis resistance in tumor cells [79]. YWHAZ encodes the 14–3-3f as an anti-apoptotic protein through the p38MAPK signaling pathway [80]. Moreover, YWHAZ has an important role in stabilization of EGFR, HER2, PKC, and b-catenin which are involved in signaling pathways, cell proliferation, and apoptosis [81–83]. It has been reported that the miR-30c increased DOX sensitivity in BC cells by targeting *YWHAZ*. There was significant miR-30c down regulation in DOX resistant breast cell lines [84]. DUSP4 belongs to the mitogen-activated protein kinase phosphatase (MKP) family that inhibits the MAPK signaling pathway [85]. It has been reported that miR-137 up regulation attenuated the DOX resistance in BC cells. MiR-137 also suppressed the EMT of breast tumor cells by *DUSP4* targeting upon DOX treatment [86].

FYN is a non-receptor tyrosine kinase involved in cell growth, apoptosis, and motility [87]. It has been shown that there was miR-381 down regulation in DOX-resistant BC cells. MiR-381 re-sensitized DOX resistant BC cells via FYN inhibition and MAPK signaling inactivation [88]. During the chemo resistance process, tumor cells are able to develop resistance mechanisms by drug efflux, inactivation of detoxification enzymes, apoptosis regulation, tumor suppressor regulation, and DNA repair induction [89–91]. ABCB1 is a drug efflux transporter involved in multidrug resistance by increasing the intracellular levels of anticancer drugs. It has been shown that the miR-302 cluster reversed the BC cells drug resistance through *ABCB1* down regulation. The miR-*302* cluster also down regulated the MEKK1 as a member of the MAPK Kinase family. Therefore, miR-302 increased DOX sensitivity in BC cells by *MEKK1* targeting and *ABCB1* inhibition [92].

### Apoptosis, cell cycle, and DNA repair

Bcl-2 interacting mediator of cell death (Bim) is a pro-apoptotic member of Bcl-2 protein family [93]. It is a key regulator of the intrinsic apoptosis pathways which directly initiates pro-apoptotic effect and induces cell apoptosis through interacting with all pro-apoptotic members of the Bcl-2 family [94, 95]. It has been reported that there were miR-181b and miR-222 up regulations in BC patients which were associated with DOX sensitivity through Bim targeting [96, 97]. The up regulation of myeloid cell leukemia 1 (MCL-1) as a pro-survival member of the Bcl-2 family, has been reported in various malignancies and shown to be correlated with a worse prognosis [98, 99]. MCL-1 enhances tumor cell survival while inhibiting their apoptosis through disrupting the normal activity of Noxa and other pro-apoptotic members of the BCL-2 family [100]. It has been shown that there was a significant miR-193b down regulation in the MCF-7/DOX resistant cells in comparison with its parental MCF-7 cells. MiR-193b increased the DOX sensitivity via *MCL-1* targeting [101].

Survivin (BIRC5) belongs to the inhibitor of apoptosis (IAP) protein family. It was initially identified as a negative regulator of apoptosis which functions through inhibiting the caspase activation; however, it is now known that the survivin has a bi functional roles in survival and cell cycle [102]. Survivin exerts its anti-apoptotic activity through blocking the CASP9 function in a complex with hepatitis B X-interacting protein (HBXIP) thereby playing a crucial role in chemo resistance. It has been reported that there were significant miR-218 down regulation in drug-resistant breast cancer cell lines. MiR-218 restored the sensitivity of drug-resistant cell lines to doxorubicin and taxol through survivin targeting and apoptosis induction [103]. External antigens induce the proliferation of CD8+ and/or CD4+ helper cells that inhibit tumor progression [104].

The programmed death-ligand 1 (PD-L1) is an immune suppressor receptor expressed in T-cell membranes that reduces the proliferation of antigen-specific T-cell in the lymph nodes and increased regulatory T cells apoptosis during immune tolerance of cancer patients [105]. PD-L1 is also involved in increased chemo resistance in BC [106, 107]. It has been reported that there were miR-3609 down regulation and PD-L1 up regulation in DOX-resistant BC cell lines compared with the sensitive cells. Therefore, miR-3609 reversed DOX resistance by *PD-L1* targeting and CD8+ T cells activation in BC cells. The miR-3609 down regulation was also correlated with poor prognosis in BC patients [108]. Peptidylprolyl isomerase A (PPIA) belongs to the peptidyl-prolyl cis/trans isomerase (PPIases) family and constitutes the cytosolic binding domain of cyclosporine A as an immunosuppressive agent. PPIA has key roles in various cellular processes such as cell proliferation, migration, apoptosis, immune regulation, and protein folding [109–111]. It has been reported that the miR-192-5p sensitized breast tumor cells to DOX and promotes apoptosis by the *PPIA* and *BCL-2* targeting. MiR-192-5p also induced JNK-mediated apoptosis and up regulated the pro-apoptotic proteins such as CASP9 and BAD [112]. The RFWD2 is an E3 ubiquitin ligase that promotes tumor growth through p53 degradation [113]. It has been reported that there was miR-214 down regulation in BC tissues which was associated with longer disease free survival. MiR-214 increased apoptosis and DOX sensitivity in BC cells via *RFWD2* targeting [114].

The findings indicated that the DOX treatment disrupted the normal cell cycle regulation by modulating the levels of miR-449 family and even its theoretically targeted genes (*CDC25A, SIRT1, GMNN, E2F1, E2F3, BCL2, CDK2*, and *CCNE2*). MiR-449 promoted DOX sensitivity by significant inhibition of cell cycle regulators including CDK2, E2F1, and E2F3 in BC cells [115]. Various mechanisms are involved in DNA repair in mammalian cells [116].

The flap endonuclease 1 (FEN1) is a critical factor during long-patch base excision repair process [117]. FEN1 has also a pivotal role during maturation of Okazaki fragments, telomere stability, and replication fork progression [118]. YY1 is a developmental transcription factor associated with cellular differentiation and proliferation [119, 120]. It has been reported that the miR-140 inhibited BC tumor progression and reduced DOX resistance through FEN1 down regulation and BER suppression. YY1 was also shown as a suppressor of FEN1 expression through miR-140 up regulation [121]. DOX-induced DNA damage activates DNA repair machinery in tumor cells. Therefore, aberrant DNA repair processes greatly influence cancer cells’ responsiveness to chemotherapy [122, 123]. It has been reported that the miR-30c was involved in DNA repair by regulation of REV1 and FANCF expressions. MiR-30c also promoted DOX-sensitivity in p53-mutant BC cells. DOX chemo resistance in p53-mutant BC cells was correlated with the miR-30c/FANCF/REV1-associated DNA damage response [124].

### Transporters

ATP-binding cassette (ABC) family of transporters are drug efflux pumps involved in tumor cells MDR [125, 126]. Multidrug resistance protein 1 (MRP1) belongs to the superfamily of ABC transporters and is encoded by the *ABCC1* gene. ABCC1 is correlated with the DOX resistance in MDR cancer cells [127, 128]. It has been observed that there was miR-134 down regulation in DOX-resistant breast tumor cells. MiR-134 significantly inhibited the cell proliferation and induced apoptosis in MCF-7/DOX cells via *ABCC1* targeting [129]. Long non-coding RNAs (LncRNAs) are a class of non-coding RNAs (> 200 nucleotides length) with pivotal roles in tumor progression and chemo resistance [130]. It has been shown that there were *linc00518* and *ABCC1* up regulations in BC tissues and cell line. DOX-resistant MCF-7 cells (MCF-7/DOX) had also increased expression levels of *linc00518* and *ABCC1* compared to parental MCF-7 cell line. *Linc00518* promoted MDR via regulating the miR-199a/ABCC1 axis in BC cells [131]. Another study has been reported that the miR-145 sensitized BC cells to DOX via *ABCC1* targeting [132].

MiR-200 family is an essential regulator of EMT process which exerts its inhibitory function on tumor cell migration and invasion through down regulating E-cadherin transcriptional repressors such as ZEB1 and ZEB2 [133, 134]. It has been reported that there was a correlation between miR-200c down regulation and a poorer response of BC patients to neoadjuvant chemotherapy. Increased sensitivity of BC to epirubicin following transfection of miR-200c mimic was achieved at least in part via the inhibitory effect of miR-200c on ABCB1 expression. There were significant different levels of miR-200c expression between clinical responders and non-responders. DOX-resistant cells had significantly increased *ABCB1* and decreased miR-200c levels compared with parental MCF-7 cells [135]. Another study also showed that the miR-451 increased DOX sensitivity of BC cells via *ABCB1* targeting [136]. It has been reported that the miR-124-3p up regulation and ABCC4 inhibition increased DOX sensitivity in BC cells. There were significant correlations between tumor size, stage, and ABCC4 up regulation. ABCC4 down regulation inhibited the cell proliferation and migration, and induced DOX sensitivity. The miR-124-3p up regulation also significantly suppressed *ABCB1* expression in MCF-7-DOX cells [137].

Uncoupling proteins (UCPs) are three structurally similar mitochondrial inner membrane transporters (UCP1/2/3) belong to the mitochondrial anion transporters family [138]. UCP-2 has a ubiquitous tissue expression and is implicated in cellular energy expenditures, mitochondrial ROS regulation, and ATP synthesis [139–142]. It has been reported that the miR-133a reduced DOX resistance in BC cells via *UCP-2* targeting [143].

### TGF-β and JAK/STAT signaling pathways

Transforming growth factor beta (TGFβ) signaling is a pivotal pathway involved in cell growth, cell differentiation, and apoptosis. This pathway is triggered by TGFβ ligands and receptors, which activates and translocates the SMAD proteins into the nucleus where they functions as transcription factors. SMAD4 is a mediator of TGF-β signaling pathway involved in the MDR of different tumors [144, 145]. It has been reported that there was significant miR-574 up regulation in Dox-resistant MCF-7 cells in comparison with parental cells. There were also increased levels of miR-574 in blood samples of advanced BC patients following chemotherapy. MiR-574 induced DOX resistance in BC cells through *SMAD4* targeting [146]. SMAD3 is essential for the TGF-β-induced EMT and mediates the mammary epithelial cells invasion [147]. It has been reported that there was a significant miR-489 down regulation in DOX-resistant BC cells. MiR-489/SMAD3 axis regulated the DOX-resistance of breast tumor cells via EMT process [148]. PBLD is a negative regulator of TGF-β1-induced EMT during tumor progression [149]. It has been reported that there was significant reduced levels of *circKDM4C* expressions in BC samples which was inversely correlated with chemo resistance through the miR-548p regulation. There was also a significant direct association between *circKDM4C* expression and overall survival. *CircKDM4C* reduced the BC progression and DOX-resistance via miR-548p sponging and PBLD activating [150].

**Fig. 3:**
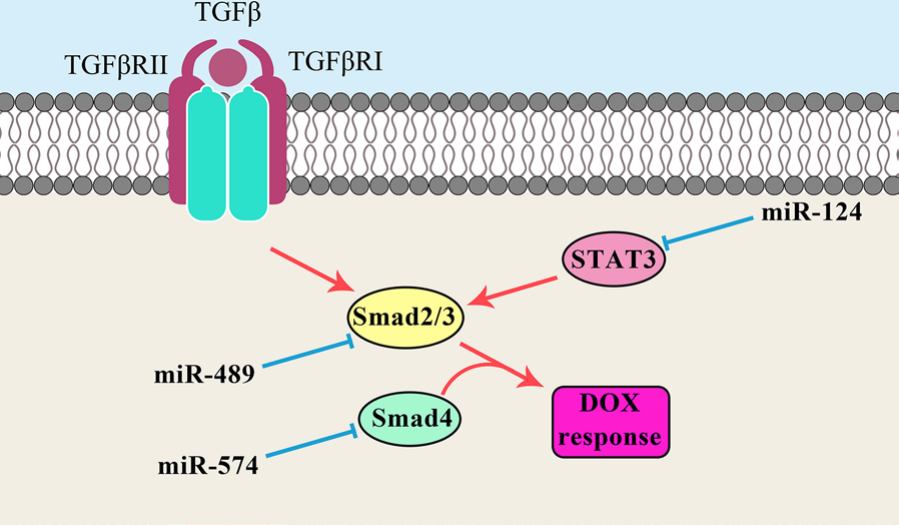
Roles of miRNAs in regulation of DOX response (resistance or sensitivity) in breast tumor cells through TGFb and JAK/STAT signaling pathways

BCSCs are a sub population of tumor cancers mainly associated with tumor relapse, chemo resistance, and poor prognosis [151]. Therefore, elimination of BCSCs seems to be effective for the solving of clinical issues like drug resistance and tumor recurrence [152]. STAT family of transcription factors regulates the multiple cellular processes. Hypoxia-inducible factor-1 (HIF-1) is the main transcription factor implicated in cellular response to hypoxia. It also regulates the different genes associated with tumor aggressiveness [153]. HIF-1 signaling is critical for the activation of NOTCH pathway that affects the EMT process [154]. It has been reported that the miR-124 was involved in DOX-resistance of BCSCs via STAT3/HIF-1 signaling pathway. DOX-resistant BCSCs showed increased levels of STAT3. STAT3 up regulated the ALDH1, OCT4, and SOX2. MiR-124 reduced the DOX-resistance in BCSCs through modulation of STAT3/HIF-1 signaling pathway [155]. Role of miRNAs in regulation of DOX response in BC through TGFb and JAK/STAT signaling pathways is illustrated in Fig. 3.

### Enzymes and structural proteins

Osteopontin (OPN) is a hydrophilic non-collagenous phosphorylated glycoprotein which is present in ECM and mediates the multiple biological functions. OPN is recognized as secreted (sOPN) or intracellular (iOPN) proteins [156]. Secreted OPN functions via interaction with the cell surface receptors including the integrin and CD44 families [157]. OPN has key roles in the diverse pathophysiological processes such as immune-mediated and inflammatory diseases as well as tissue and bone remodeling [158, 159] and is also implicated in tumor progression, metastasis, and angiogenesis [160]. It has been reported that there was a significant miR-181c down regulation in BC cells. MiR-181c suppressed the breast tumor cell proliferation and invasion while promoted DOX sensitivity. There was an inverse correlation between the miR-181c and *OPN* expression levels which was associated with the DOX response, metastasis, and BC patients’ overall and disease-free survival. Moreover, miR-181c inhibited the EMT of BC cells via vimentin and N-cadherin down regulations and E-cadherin up regulation [161].

Anterior gradient 2 (AGR2) belongs to the protein disulfide isomerases (PDIs) family which plays an important role in mammary epithelial proliferation, lobuloalveolar development, and protein folding [162, 163]. AGR2 up regulation early in tumorigenesis or in response to anti-hormone treatment is associated with intrinsic or acquired resistance to therapies in ER-positive breast cancers, respectively [164]. DOX-resistant BC cells were observed to have AGR2 over expression. Up regulated and down regulated AGR2 were correlated with increased and reduced DOX-sensitivity, respectively. It was also found that miR-135b-5p enhanced the DOX-sensitivity of BC cells through *AGR2* targeting. MiR-135b-5p/AGR2 axis was suggested as an important pathway responsible for DOX-sensitivity in BC cells [165].

Nicotinamide phosphoribosyl transferase (NAMPT) as an important factor involved in NAD synthesis has pivotal roles in the immune response and metabolism [166, 167]. NAD is a substrate for the sirtuin deacetylase in transcriptional regulation of other genes [168]. NAMPT also promotes BC cell proliferation through stimulation of ER activity [169]. Moreover, NAMPT up regulation can be associated with DOX resistance in BC patients [170]. It has been reported that there was significant miR-154 down regulation in BC cell lines compared with normal mammary cells. There was an inverse association between the *NAMPT* and miR-154 expressions in BC cells. MiR-154 sensitized the BC cells to DOX through *NAMPT* targeting [171]. Stathmin1 (STMN1) is a microtubule-destabilizing factor involved in the regulation of cytoskeleton and microtubule dynamics [172]. STMN1 enhances the microtubule depolymerization via sequestering free tubulins [173, 174]. MiR-770 was significantly down regulated in chemo-resistant BC tissues. It also increased the DOX-sensitivity through *STMN1* targeting [175].

## Conclusions

DOX is one of the common first line chemotherapeutic drugs used for BC treatment; however there is a high ratio of DOX resistance among the BC patients. Since, DOX has severe side effects; it is required to distinguish the non DOX-responders from responders and also clarify the molecular mechanisms involved in DOX resistance to provide novel efficient therapeutic modalities to improve the clinical outcomes of BC patients. MiRNAs are important factors involved in drug resistance through regulation of drug efflux, DNA repair, cell cycle, and signaling pathways. They are also non-invasive and more stable factors compared with mRNAs. This review highlights the miRNAs as pivotal regulators of DOX resistance in breast tumor cells. Moreover, present review paves the way of introducing a non-invasive panel of prediction markers for DOX response among BC patients.

## Data Availability

The datasets used and/or analyzed during the current study are available from the corresponding author on reasonable request.

## Abbreviations

BC: Breast cancer
DOX: Doxorubicin
miRNAs: MicroRNAs
PR: Progesterone receptor
ER: Estrogen receptor
TNBC: Triple-negative breast cancer
ADR: Adriamycin
MDR: Multidrug resistance
MRP1: Multiple resistance protein-1
UTR: Untranslated region
BCSCs: Breast cancer stem cells
FZD: Frizzled
ECM: Extra cellular matrix
EMT: Epithelial mesenchymal transition
Daam1: Dishevelled-associated activator of morphogenesis 1
CSCs: Cancer stem cells
PRKD1: Protein kinase D1
PTEN: Phosphatase and tensin homolog
GSK-3β: Glycogen Synthase Kinase 3β
TrkB: Tropomyosin receptor kinase B
MAPK: Mitogen-activated protein kinase
MKP: Mitogen-activated protein kinase phosphatase
Bim: Bcl-2 interacting mediator of cell death
MCL-1: Myeloid cell leukemia 1
IAP: Inhibitor of apoptosis
HBXIP: Hepatitis B X-interacting protein
PD-L1: Programmed death-ligand 1
PPIA: Peptidylprolyl isomerase A
PPIases: Peptidyl-prolyl cis/trans isomerase
ABC: ATP-binding cassette
MRP1: Multidrug resistance protein 1
LncRNAs: Long non-coding RNAs
UCPs: Uncoupling proteins
TGFβ: Transforming growth factor beta
HIF-1: Hypoxia-inducible factor-1
OPN: Osteopontin
sOPN: Secreted Osteopontin
iOPN: Intracellular Osteopontin
AGR2: Anterior gradient 2
PDIs: Protein disulfide isomerases
NAMPT: Nicotinamide phosphoribosyl transferase
STMN1: Stathmin1
